# Total Sleep Deprivation Triggers Greater Activation in the Parietal Brain in the Visual Working Memory Updating Processes: An Event-Related Potentials Study

**DOI:** 10.3389/fnins.2022.736437

**Published:** 2022-03-16

**Authors:** Tao Song, Ke Yu, Letong Wang, Lin Xu, Mengmeng Xu, Ziyi Peng, Cimin Dai, Haiteng Wang, Tianyi Yang, Yongcong Shao, Xiaoming Wang, Jing Lv

**Affiliations:** ^1^School of Psychology, Beijing Sport University, Beijing, China; ^2^Department of General Practice, The General Hospital of Western Theater Command, Chengdu, China; ^3^Tianjin Institute of Environmental and Operational Medicine, Tianjin, China; ^4^Department of Psychology, The Second Medical Center, Chinese PLA General Hospital, Beijing, China

**Keywords:** sleep deprivation, working memory, event-related potentials, P3, compensatory neural activity

## Abstract

Working memory functions are known to be altered after total sleep deprivation (TSD). However, few studies have explored the deficits of working memory updating (WMU) after TSD, or the underlying electrophysiological mechanisms of these alterations. In the current exploratory study, we enrolled 14 young male volunteers who performed two kinds of WMU tasks—spatial and object two-back tasks—with simultaneous electroencephalography recordings under two sleep conditions: a normal sleep baseline condition and after 36 h of TSD. Repeated-measures analysis of variance showed that compared with those at baseline, the rates of correct responses in the WMU tasks decreased significantly after TSD. Analysis of event-related potentials revealed that the average amplitude of P3 components decreased significantly in the frontal and central brain regions and increased significantly in the parietal brain regions. Our findings suggest that TSD damages WMU behavior, impairs cognitive functions in the frontal and central brain regions, and triggers greater activation in the parietal brain regions. This is the first study to report the existence of event-related compensatory neural activity. This event-related compensatory effect may provide a new perspective for understanding the mechanisms underlying the influences triggered by sleep loss.

## Introduction

With smart devices changing the style of nightly leisure-time activities and work schedules, an increasing number of people experience sleep restriction. There is growing evidence that sleep influences the risk of cardiovascular disease (St-Onge and Zuraikat, 2019). In particular, personnel in many fields of work are influenced by shortened sleep, which puts them at higher risk when working. For instance, shortened sleep is an important cause of industrial and transport-related accidents (Philip and Akerstedt, 2006). Therefore, exploring why shortened sleep affects cognitive functions is essential to reduce the harm caused by it. Total sleep deprivation (TSD) represents a comprehensive end of shortened sleep and is a convenient metric to thoroughly examine how shortened sleep impairs cognition (Lowe et al., 2017). Therefore, information about the effects of shortened sleep on neurocognitive functions is primarily obtained from acute TSD experimental studies.

Working memory is the basis for higher-order cognitive functions. It refers to a narrow-capacity system that allows temporary storage and active manipulation of information necessary for more complex tasks (Baddeley, 2012). Baddeley (2012) proposed and revised the working memory model, which includes the central executive and the episodic buffer, visuospatial sketchpad, and phonological loop subsystems. The central executive controls and supervises the processing of information entering the phonological loop or visuospatial sketchpad. The central executive is an attentional system composed of several executive functions (Baddeley, 2012), such as working memory updating (WMU) and inhibition control. The WMU function, coordinated by the central executive, is the act of updating the current status of a representation of a pattern in working memory to accommodate new input (Morris and Jones, 1990). Numerous studies have revealed that TSD impairs executive functions. Aidman et al. (2018) carried out a TSD study including running letters test, spatial Stroop test, and predictable switching digits task, and they showed that TSD damages three kinds of executive functions, that is, working memory capacity, inhibitory control, and cognitive flexibility. In addition, Cain et al. (2011) found TSD influences performance on the Stroop task by an overall increase in response time. Honn et al. (2018) revealed that TSD impairs feedback blunting in a cognitive flexibility task. In addition, a few studies have shown that cognitive function in females is less susceptible to the influence of TSD (Binks et al., 1999; Corsi-Cabrera et al., 2003), which may be interpreted by the gonadal hormone status (Hajali et al., 2019). Taken together, these studies suggest that executive functions are negatively affected by TSD.

The mechanisms of the aforementioned TSD-induced alterations have been explored to a certain degree *via* functional magnetic resonance imaging (fMRI) studies. Reduced dorsolateral prefrontal cortex and parietal activity have been shown to correlate with detrimental effects on working memory (Chee and Choo, 2004; Habeck et al., 2004; Choo et al., 2005; Chee and Chuah, 2007; Lythe et al., 2012). Alterations in thalamic activity and functional connectivity predict deficits in the performance of working memory under TSD conditions, most likely due to the important role of the cortical arousal of the thalamus (Krause et al., 2017). Many studies reported that functional connectivity is attenuated after TSD as well. For instance, the connectivities between the right middle occipital gyrus/precuneus and the left inferior/middle temporal gyrus or left parahippocampal/fusiform gyrus are attenuated after TSD (Zhang et al., 2019a). Moreover, greater risk-taking is paralleled by less activation of the dorsolateral prefrontal cortex in cognitive control processing, greater insula activation in reward processing, and weakened functional connectivity between the dorsolateral prefrontal cortex and affective regions, including the ventral striatum and insula in reward processing (Telzer et al., 2013). Wang et al. (2014, 2020) reported that TSD impairs functional connectivity within the default network, which may explain why TSD triggers mood disorders. Electroencephalography (EEG) studies also provided evidence for interpreting TSD-induced alterations. Using a stop-signal task with simultaneous EEG, Kusztor et al. (2019) dissociated three subcomponents of cognitive control: sustained attention, automatic bottom-up processing, and strategic top-down control, and found that TSD caused a decline in sustained attention and reduced P3 and P-e amplitudes. These results suggested a progressive collapse of the top-down control subcomponent rather than that of the other two subcomponents. Gibbings et al. (2020) found more intense alpha bursts result in sleep-related deficits in vigilant attention, which may be the fundamental mechanism of the detrimental effect induced by TSD. Taken together, accompanied by damaged executive functions, brain morphology is also negatively affected by TSD (Dai et al., 2018).

Surprisingly—considering the important role of WMU in cognition—only a few studies have explored whether TSD changes WMU function. There is a wide gap in the number of study topics related to different central executive functions. Cognition flexibility (Couyoumdjian et al., 2010; Honn et al., 2018) and inhibition control (Renn and Cote, 2013; Slama et al., 2018; Gosselin et al., 2019) after TSD have been studied extensively, whereas WMU has not. Gerhardsson et al. (2019) found that TSD attenuated the accuracy rather than the speed of working memory (updating). However, compared with the typical WMU paradigm, the task they used—the emotional n-back task, which includes TSD, emotion, and WMU measures—was so complicated that the conclusions were not reliable. Another study reported that TSD did not alter WMU measures in the n-back task (Slama et al., 2018). However, in the two-back task of the study, participants were asked to respond only when the current stimulus matched the stimulus presented two steps earlier in the task sequence, which is different from the typical WMU paradigm. Therefore, the results of this study were also inconclusive. Zhang et al. (2019b) argued that TSD damaged WMU processing, as measured *via* the classical pronunciation two-back task. It is still unclear whether visual WMU is influenced by TSD; therefore, we designed the present study including two visual WMU tasks to fill this knowledge gap. Based on the two types of visual working memory, we designed two types of visual WMU tasks: spatial and object WMU tasks. Since being developed by Kirchner (1958), the n-back task has been widely used for experimental research on working memory yield. It can successfully predict individual differences in higher cognitive functions such as fluid intelligence, especially when the levels of loads in the task are higher (Jaeggi et al., 2010). A meta-analysis verified that the reliability of the n-back task was high in fMRI studies (Owen et al., 2005). Using latent factor analyses, Schmiedek et al. (2014) demonstrated that n-back tasks are effective indicators of working memory. The N-back task requires participants to decide whether the current stimulus is as same as (match) or different from (mismatch) the one that presented n-steps previously in the stimulus sequence. Since the n-back task is frequently used in WMU-related studies (McElree, 2001; Ecker et al., 2010; Vilà-Balló et al., 2018; Salmi et al., 2019), we used visual two-back tasks in the current study.

Compensatory adaptation can offset insufficient cognitive functions (e.g., Goshen et al., 2011; Kant and Jha, 2019). Since Drummond et al. (2000) first reported the dynamic, compensatory adaptations in the prefrontal cortex and parietal lobes after TSD, previous research on TSD suggests the existence of compensatory neural activity that can enable partial recovery of working memory performance. For instance, enhanced functional connectivity is reported within the ventral default mode network, as well as between two subsystems of the default mode network, which correlates with behavioral performance after TSD (Chen et al., 2018). Functional connectivity between navigation-related brain structures increases during relearning in the extended environment after TSD, which represents the use of compensatory brain resources (Deantoni et al., 2021). The increased functional connectivity between the thalamus and precuneus under TSD conditions is paralleled with greater recovery of working memory performance (Yoo et al., 2007; Li et al., 2016). Thalamic compensatory responses recruited by TSD can counter the detrimental effect to support thalamocortical function (Muto et al., 2012). Increased voxel-mirrored homotopic connectivity might reflect the compensatory activity of the bilateral thalamus to prevent cognitive performance deterioration after TSD (Zhu et al., 2016). The small-world property is significantly increased under TSD conditions, reflecting a possible compensatory activity of the human brain (Liu et al., 2014). In addition to TSD, sleep restriction also triggers compensatory neural activity. Alsameen et al. (2021) found that sleep restriction results in greater medial prefrontal activation for the most difficult working memory task condition. Overall, the existence of compensatory neural activity is accurate. However, the processing of compensatory neural activity in the time domain is still unclear. In other words, there may be an event-related compensatory neural activity within 1 s or less during the WMU processes. In the current study, we used visual two-back tasks before and after TSD, and simultaneous EEG recordings to examine it. Further, considering that the prefrontal cortex is involved in executive functions like WMU, we hypothesized that the event-related compensatory neural activity would occur in the parietal brain.

Apart from examining event-related compensatory neural activity in the parietal brain, we also investigated the alterations in the WMU process after TSD in the time domain to examine which stage of the WMU processes was affected by TSD: the primary selection of information or the allocation of attentional resources. Among the approved event-related potential (ERP) components triggered by each event—P1, N1, P2, N2, P3, and LPP—the P2 and P3 components were considered suitable for the current study. Modulation of the P2 component has been reported in many WMU tasks (McEvoy et al., 1998; Rämä et al., 2000; Lenartowicz et al., 2010; Luu et al., 2014; Vilà-Balló et al., 2018; Salmi et al., 2019); it is commonly associated with primary top-down attentional control processing (Freunberger et al., 2007) and useful selection of information at the sensory cortices (Crowley and Colrain, 2004). Modulation of the P3 component is one of the most repeated findings in ERP studies in the field of WMU; in particular, the P3b subcomponent is related to downstream cognitive control processing and allocation of attentional resources (Donchin et al., 1986; Lenartowicz et al., 2010; Daffner et al., 2011; Vilà-Balló et al., 2018; Salmi et al., 2019). Since TSD is more detrimental to cognitive functions, which depends more on mental effort, rather than more automatic control processes (Kusztor et al., 2019), we hypothesized that TSD would affect the allocation of attentional resources, instead of the primary selection of information, during WMU processes.

## Materials and Methods

### Participants

Fourteen young male volunteers (25.9 ± 2.3 years) participated in the current study. All participants were right-handed and had normal or corrected normal vision. They had no history of neurological or mental disorders, and their Raven test scores were higher than 110. The experimental scheme was approved by the Ethics Committee of the Fourth Military Medical University and Beihang University. All participants maintained healthy sleep habits (PSQI score < 5, Buysse et al., 1989). For half a month before the beginning of the TSD experiment, they were instructed to sleep before 10 p.m. and wake up after 6 a.m. every day to ensure adequate sleep for 7–9 h. All participants declared that they did not have a habit of smoking cigarettes or drinking alcohol or coffee and had not taken any medication within 48 h before the experiment. Each participant signed an informed consent form without any doubts or objections before the beginning of the experiment.

### Experimental Design

**FIGURE 1:**
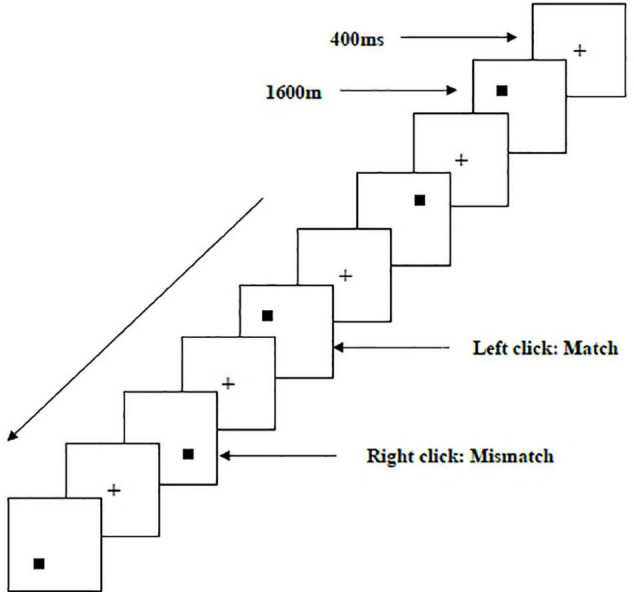
Schematic diagram of the spatial working memory updating task.

**FIGURE 2:**
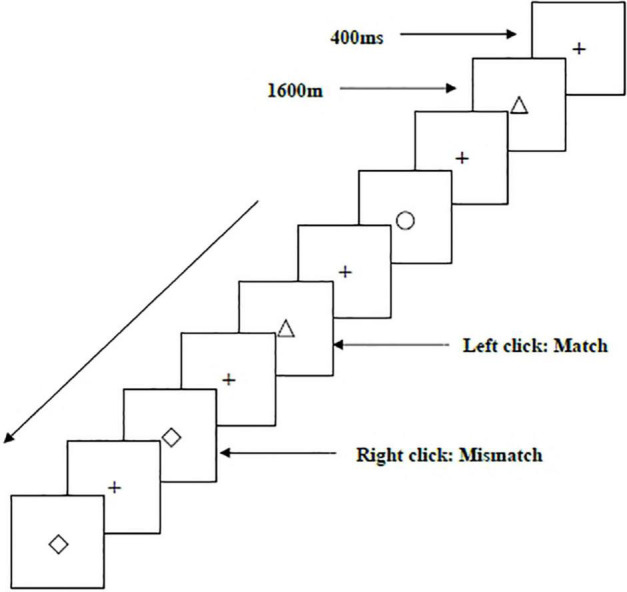
Schematic diagram of the object working memory updating task.

Two types of WMU tasks—a spatial two-back task (Figure 1) and an object two-back task (Figure 2)—were presented to all participants. The stimulus materials for the two tasks were small black squares and 12 geometric figures, respectively. A black stimulus material was presented on a white background with an approximate visual angle of subtending 1.5° × 1.5° and a width and height of 2.0 cm each. Each stimulus sequence comprised 122 trials. In each trial, a fixation point marked by a black “+” appeared for 400 ms, followed by an objective stimulus material presented for 1,600 ms (Salmi et al., 2019). When the two-back target was consistent with the stimulus being presented (match), each participant clicked the left mouse button as required; when the two were inconsistent (mismatch), participants clicked the right mouse button. The order of stimulation sequence for each participant was randomized. Before the start of each stimulation sequence, the experimenter informed the participant about the type of task to be performed.

### Experimental Procedures

Two participants conducted the experiment simultaneously. The participants arrived at the laboratory the day before the experiment and slept in the laboratory at night (Yoo et al., 2007). Before the experiment, all participants practiced the two types of WMU tasks until they reached an accuracy of 90%. The following day, the participants completed two WMU tasks with simultaneous EEG recordings (baseline) from 7:30 to 8:30 a.m. (Wu et al., 2021). After 36 h of TSD, from 7:30 to 8:30 p.m. the next day (Wu et al., 2021), they completed similar WMU tasks with the second simultaneous EEG recording (TSD). The participants were instructed to stay awake throughout the TSD period, and were prohibited from taking concentrated inhibitory or irritating drugs during the entire experiment. Paramedics accompanied and observed the participants and reminded them to remain awake throughout the TSD period.

### Behavioral Data Analysis

Behavioral data and accuracy rates in the two WMU tasks under baseline and TSD conditions were recorded for analysis. The analyses—repeated measures analysis of variance (ANOVA) and Bonferroni *post-hoc* analysis—were performed using the SPSS software (IBM SPSS Statistics for Windows, Version 22.0. IBM Corp., Armonk, NY, United States). The statistical results are presented as the mean ± standard deviation. The main effects and the interactions between sleep conditions (baseline and TSD) and task types (spatial WMU task and object WMU task) were analyzed.

### Electroencephalography Recordings and Preprocessing

EEG was recorded continuously from 32 electrodes using a SynAmps2 amplifier (Compumedics Neuroscan, Victoria, Australia) with a sampling rate of 1,000 Hz. The electrodes were mounted on an elastic cap according to the 10–20 system standard positions. The impedance of the electrode was reduced and maintained below 5 kΩ. The horizontal/vertical eye movements were monitored with an electrode placed above/below the left eye. The on-line reference electrodes were placed on the bilateral mastoids.

The raw EEG data were preprocessed offline in MATLAB R2017a (The MathWorks, Inc., Natick, Massachusetts, United States)^1^ using the EEGLAB2020_0 toolbox (Delorme and Makeig, 2004). The sampling rate was reduced to 250 Hz, and the average reference was used as re−reference. A band-pass filter of 0.1–40 Hz was used *via* a 4th order Butterworth filter with a frequency slope of 24 dB/oct in the ERPLAB plugin ^2^ (Lopez-Calderon and Luck, 2014). After independent component analysis, components symbolizing eye movement and inordinate muscle activity were identified by the ADJUST plugin (an auxiliary tool for artifact rejection,^3^ Mognon et al., 2011) and removed. Epochs with a length of 1,000 ms ranging from −200 ms to 800 ms with respect to the onset of the stimuli were then extracted from the continuous EEG data. The stimuli-locked ERPs were baseline-corrected in the range of −200 ms to 0 ms before stimulus onset. We preprocessed raw EEG signals and extracted features following the standard processing procedures (Tripathi et al., 2018; Zhang et al., 2020).

### Electroencephalography Data Analysis

The characteristics of ERP components were extracted using the Letswave7 toolbox^4^ after averaging and calculating using only the corrected responses. Two components from the stimulus trials, P2 (100–250 ms) and P3 (250–450 ms), were identified, extracted, and quantified. The grand-average mean amplitudes of the P2 components were calculated separately in the frontal (F3, Fz, F4) and central (C3, Cz, C4) regions. The grand-average mean amplitudes of the P3 components were calculated separately in the frontal (F3, Fz, F4), central (C3, Cz, C4), and parietal (P3, Pz, P4) regions. These regions have been shown to display altered activation following sleep deprivation (Gevins et al., 1996; Casement et al., 2006; Verweij et al., 2014).

A 2(sleep conditions: baseline, TSD) × 2(task types: spatial WMU task, object WMU task) repeated-measures ANOVA—including Greenhouse–Geisser corrections for non-sphericity and Bonferroni *post-hoc* tests—was used to analyze the mean amplitudes of the P2 and P3 components. The main effects and the interactions between sleep conditions and task types were separately analyzed in different regions (frontal, central, and parietal).

## Results

### Behavioral Results

The results of the behavioral experiments are shown in Table 1.

**TABLE 1:** Mean accuracy (and standard deviations; %) in the working memory updating tasks before (baseline) and after total sleep deprivation (TSD).

	Baseline	TSD
Spatial WMU task	92.01 ± 4.60	86.02 ± 11.27
Object WMU task	88.86 ± 6.14	82.14 ± 12.99

Repeated-measures ANOVA of accuracy revealed a significant main effect of sleep conditions [*F*_(1, 13)_ = 5.36, *p* = 0.038, partial η^2^ = 0.29, 1-β = 0.57], suggesting that accuracy was higher at baseline than after TSD. The main effect of task types was also significant [*F*_(1, 13)_ = 5.07, *p* = 0.042, partial η^2^ = 0.28, 1-β = 0.55], suggesting that the accuracy of the spatial WMU task was higher than that of the object WMU task. There was no significant interaction between sleep conditions and task types [*F*_(1, 13)_ = 0.09, *p* = 0.764, partial η^2^ = 0.01, 1-β = 0.06], suggesting that the differences in accuracy between spatial and object WMU tasks were equally distributed across sleep conditions.

### Event-Related Potential Results

**FIGURE 3:**
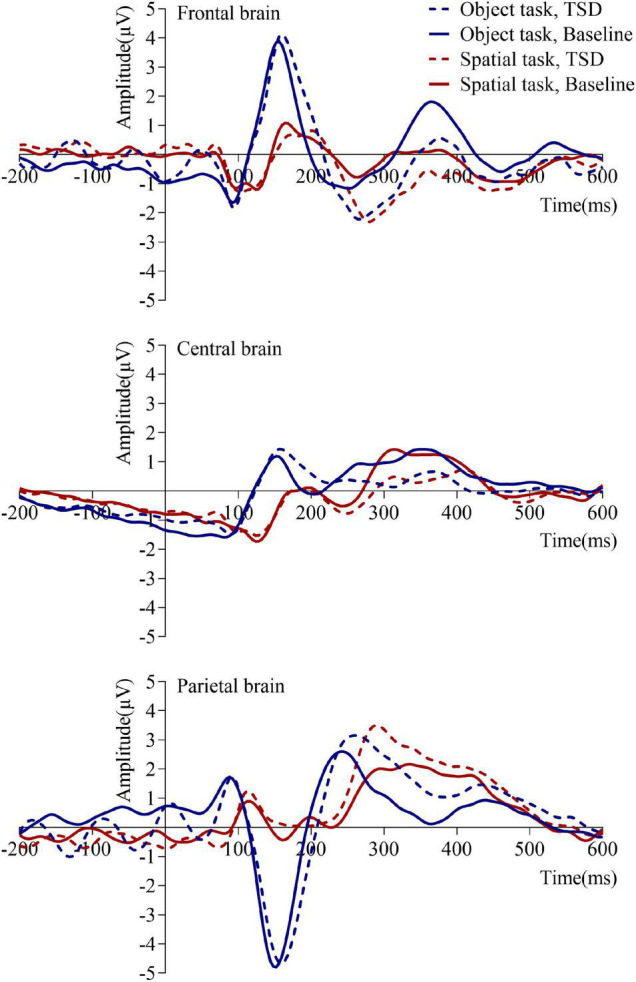
Mean amplitudes of event-related potentials under baseline and 36 h total sleep deprivation (TSD) conditions for correct responses to two working memory updating tasks. The brain regions are ordered from top to bottom as follows: the frontal brain (F3, Fz, F4), central brain (C3, Cz, C4), and parietal brain (P3, Pz, P4).

**FIGURE 4:**
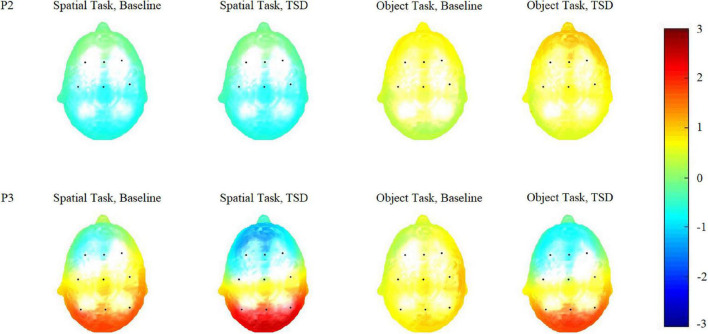
Topographic maps of the P2 (100–250 ms) and P3 (250–450 ms) components triggered by the two working memory updating tasks in different sleep conditions [baseline and after total sleep deprivation (TSD)].

Table 2 and Figures 3, 4 show that after stimulus onset, a P2 component with frontal and central distributions was observed, followed by a P3 component with a global distribution.

**TABLE 2:** Mean amplitudes (and standard deviations; μV) triggered by the working memory updating tasks before (baseline) and after total sleep deprivation (TSD).

		Baseline		TSD	
		P2	P3	P2	P3
Task1	Frontal	0.02 ± 1.27	-0.27 ± 1.44	−0.08 ± 1.14	−1.26 ± 1.43
	Central	−0.61 ± 0.5	0.83 ± 1.02	−0.64 ± 0.43	0.26 ± 0.75
	Parietal	−	1.77 ± 1.74	−	2.46 ± 1.88
Task2	Frontal	0.85 ± 1.38	0.34 ± 1.37	1.19 ± 1.41	−0.75 ± 1.29
	Central	0.3 ± 0.84	0.95 ± 1.14	0.54 ± 0.88	0.29 ± 0.92
	Parietal	−	0.86 ± 1.73	−	1.83 ± 1.83

### P2 Components

Repeated-measures ANOVA of mean amplitudes of the P2 components in the frontal region revealed a significant main effect of task types [*F*_(1, 13)_ = 27.27, *p* < 0.001, partial η^2^ = 0.68, 1-β = 1.00], suggesting that the mean amplitudes triggered by the spatial WMU task were lower than those triggered by the object WMU task. The interaction between sleep conditions and task types was significant [*F*_(1, 13)_ = 4.71, *p* = 0.049, partial η^2^ = 0.27, 1-β = 0.52], suggesting that the mean amplitudes triggered *via* the two WMU tasks were different across sleep conditions. There was no significant main effect of sleep conditions [*F*_(1, 13)_ = 0.63, *p* = 0.441, partial η^2^ = 0.05, 1-β = 0.11], suggesting that there were no overall differences in mean amplitudes of the P2 components in the frontal region when responses were combined across two WMU tasks. Subsequent simple effect analyses were also not significant [spatial WMU task: *F*_(1, 13)_ = 0.42, *p* = 0.529, partial η^2^ = 0.03; object WMU task: *F*_(1, 13)_ = 2.84, *p* = 0.116, partial η^2^ = 0.18].

Repeated-measures ANOVA of mean amplitudes of the P2 components in the central region revealed a significant main effect of task types [*F*_(1, 13)_ = 41.66, *p* < 0.001, partial η^2^ = 0.76, 1-β = 1.00], suggesting that the mean amplitudes triggered by the spatial WMU task were lower than those triggered by the object WMU task. There was no significant main effect of sleep conditions [*F*_(1, 13)_ = 1.01, *p* = 0.332, partial η^2^ = 0.07, 1-β = 0.15], suggesting that there were no overall differences in mean amplitudes of the P2 components in the central region when responses were combined across the two WMU tasks. There was no significant interaction between sleep conditions and task types [*F*_(1, 13)_ = 1.98, *p* = 0.183, partial η^2^ = 0.13, 1-β = 0.26], suggesting that the differences in mean amplitudes triggered by the two WMU tasks were equally distributed across sleep conditions.

### P3 Components

Repeated-measures ANOVA of mean amplitudes of the P3 components in the frontal region revealed a significant main effect of task types [*F*_(1, 13)_ = 21.34, *p* < 0.001, partial η^2^ = 0.62, 1-β = 0.99], suggesting that the mean amplitudes triggered by the spatial WMU task were lower than those triggered by the object WMU task. The main effect of sleep conditions was significant [*F*_(1, 13)_ = 18.03, *p* = 0.001, partial η^2^ = 0.58, 1-β = 0.98], suggesting that the mean amplitudes of the P3 components under the TSD condition were lower than those at baseline. There was no significant interaction between sleep conditions and task types [*F*_(1, 13)_ = 0.28, *p* = 0.604, partial η^2^ = 0.02, 1-β = 0.08], suggesting that the differences in mean amplitudes of the P3 components triggered *via* the two WMU tasks in the frontal region were equally distributed across sleep conditions.

Repeated-measures ANOVA of mean amplitudes of the P3 components in the central region revealed that the main effect of sleep conditions was significant [*F*_(1, 13)_ = 12.85, *p* = 0.003, partial η^2^ = 0.50, 1-β = 0.91], suggesting that the mean amplitudes were lower under TSD conditions than at baseline. There was no significant main effect of task types [*F*_(1, 13)_ = 0.22, *p* = 0.651, partial η^2^ = 0.02, 1-β = 0.07], suggesting that there were no overall differences in mean amplitudes when responses were combined across two sleep conditions. Moreover, there was no significant interaction between sleep conditions and task types [*F*_(1, 13)_ = 0.36, *p* = 0.558, partial η^2^ = 0.03, 1-β = 0.09], suggesting that the differences in mean amplitudes of the P3 components triggered *via* the two WMU tasks in the central region were equally distributed across sleep conditions.

Repeated-measures ANOVA of mean amplitudes of the P3 components in the parietal region revealed a significant main effect of sleep conditions [*F*_(1, 13)_ = 14.26, *p* = 0.002, partial η^2^ = 0.52, 1-β = 0.94], suggesting that the mean amplitudes were higher in TSD conditions than at baseline. The main effect of task types was significant [*F*_(1, 13)_ = 34.87, *p* < 0.001, partial η^2^ = 0.73, 1-β = 1.00], suggesting that the mean amplitudes triggered by the spatial WMU task were higher than those triggered by the WMU task. There was no significant interaction between sleep conditions and task types [*F*_(1, 13)_ = 1.25, *p* = 0.284, partial η^2^ = 0.09, 1-β = 0.18], suggesting that the differences in mean amplitudes of the P3 components triggered *via* the two WMU tasks in the parietal region were equally distributed across sleep conditions.

Based on the differences in the P3 components (250–450 ms), we plotted topographic maps (Figure 4) to visualize the responses triggered *via* the two WMU tasks in different brain regions. Compared with the brain responses triggered by the spatial WMU task at both baseline and in TSD conditions, those triggered by the object WMU task were stronger in the frontal and central regions and weaker in the parietal region. However, compared with those at baseline, the brain responses triggered by both WMU tasks under TSD conditions were weaker in the frontal and central regions and stronger in the parietal region. These topographic map findings are consistent with the above results.

## Discussion

The most important finding of the current study was the selective alteration of P3 amplitudes after TSD, reflecting TSD impairs cognitive functions in the frontal and central brain regions and triggers the event-related compensatory neural activity in the parietal brain regions.

The behavioral results of the current study corresponded with those of previous studies and indicated that a decrease in accuracy reflected impaired WMU function after TSD. Rabat et al. (2019) found that TSD was associated with deficits and fewer correct responses in a two-back task. In another WMU study (information replacement of working memory), behavioral performance in the classical pronunciation two-back task was attenuated under TSD conditions (Zhang et al., 2019b). Despite the shortcoming that the emotional n-back task used was not a typical WMU paradigm, the results still showed that TSD was detrimental to accuracy (Gerhardsson et al., 2019). McMahon et al. (2018) found that TSD undermined performance in a one-back working memory task. Another study also showed that TSD did not affect WMU measures in the n-back task (Slama et al., 2018), which is contrary to our findings. We suggest that the requirements of a task response resulted in this inconsistency. In the study by McMahon et al. (2018), participants were instructed to respond when the current stimulus matched the stimulus presented two steps earlier in the task sequence, which was an easier task than the one used in the current study. The behavioral results of the current study demonstrate that TSD damages the visual WMU function.

The P2 amplitudes of visual WMU tasks in the frontal and central regions neither increased nor decreased under the two sleep conditions. The P2 component is linked to the early stage of active information processing and useful selection of information in the sensory cortices (Crowley and Colrain, 2004). Freunberger et al. (2007) suggested that the P2 component was associated with the early cognitive matching system, which compared information obtained from sensory input and stored memory. Compared with those at baseline, the resources used for sensory inputs were reduced under TSD conditions, and the sensory system could still complete the processing of information collection. This finding is in line with those of many previous studies. Jugovac and Cavallero (2012) separated three attentional networks (alerting, orienting, and executive control) to evaluate the impact of TSD and found that it selectively decreased the efficacy of executive control rather than those of alerting and orienting. Another study reported that, compared with the latter stages of cognitive functions, the initial stage of automatic processing had higher resilience to TSD (Kusztor et al., 2019). The current findings regarding the P2 component provide new evidence for these hypotheses and help explain the underlying electrophysiological mechanisms.

The P3 amplitude of visual WMU tasks in parietal regions significantly increased after TSD, which was the first experimental discovery. We suggest that this interesting finding exhibits an event-related compensatory effect elicited *via* TSD in the transient sub-second period. Alongside brain imaging studies demonstrating that reductions in prefrontal and parietal cortex correlate with detrimental effects on working memory (Chee and Choo, 2004; Choo et al., 2005; Chee and Chuah, 2007; Lythe et al., 2012), many studies have shown that compensatory neural activity can enable partial recovery of cognitive functions. Drummond et al. (2000) first showed that there were dynamic, compensatory changes in prefrontal cortex and parietal lobe activation during the processing of verbal learning under TSD conditions. Subsequently, many researchers have provided evidence concerning compensatory effects from other aspects. An increased functional connectivity between the thalamus and the precuneus under TSD conditions was found, which provided evidence of greater recovery of working memory performance (Yoo et al., 2007; Li et al., 2016). An increased voxel-mirrored homotopic connectivity may reflect the compensatory adaptation of bilateral brain areas, especially the bilateral thalamus, to prevent deterioration of cognitive performance after TSD. Moreover, enhanced functional connectivity was reported within the ventral default mode network as well as between two subsystems of the default mode network, which correlated with behavioral performance after TSD (Chen et al., 2018), and the functional connectivity between the dorsal attention network and default mode network significantly increased after TSD (Dai et al., 2020). In addition, Liu et al. (2014) revealed that TSD significantly enhanced the small-world property of the human brain, exhibiting a possible compensatory effect evoked by TSD. The above findings from block-designed fMRI, rest-state fMRI, or rest-state EEG experimental studies support the existence of compensatory neural activity after TSD originating. Along with stateful compensatory neural activity, the present study suggests the existence of compensatory neural activity related to stimuli in the transient sub-second period, which may be called event-related compensatory neural activity. Although the spatial resolution of EEGs was lower than that of fMRI, it could still be preliminarily hypothesized that the event-related compensatory neural activity in visual WMU processes after TSD is located in the parietal regions.

The P3 amplitude of the visual WMU task in the frontal and central regions significantly decreased after TSD, which is consistent with many previous studies. Zhang et al. (2019b) reported that the decreased P3 amplitude after TSD was part of the electrophysiological mechanisms related to decreased WMU processing. Renn and Cote (2013) demonstrated that in the Go/No-Go task, P3 amplitudes were weaker in Go trials, which indicated impaired attention caused by TSD. Kusztor et al. (2019) reported that TSD resulted in reduced P3 amplitudes and declined sustained attention, manifesting a gradual breakdown of top-down control. In an auditory study (Muller-Gass and Campbell, 2019), participants were instructed to concentrate on a duration classification task under both normal sleep and TSD conditions. The P3 component is associated with the allocation of attentional resources (Donchin et al., 1986), and the P3 potential is known to be a sensitive cognitive measure of sleep deprivation (Morris et al., 1992). Gevins et al. (1996) demonstrated that WMU P3 components appeared first in the posterior prefrontal regions and then in the posterior parietal regions, which is consistent with the results of the current study. TSD had a significant effect on the attention-dependent duration detection task, resulting in worse performance and attenuated P3 components. According to a previous summary, TSD affected tasks depend on sustained selective attention (Killgore, 2010); therefore, it was not surprising that the mean amplitudes of P3 components in the frontal and central regions decreased in the current study. In addition, WMU training altered the P3 components, and this should be considered when interpreting the differences in P3 components between sleep conditions. While P3 amplitudes were enhanced after WMU training (Pergher et al., 2018; Salmi et al., 2019), those in the frontal and central regions were attenuated after TSD, indicating lower allocation to active sustained attention in WMU processes after TSD than during normal sleep baseline conditions.

The changed process of WMU after TSD can be clarified by combining the findings related to the two components, P2 and P3. In the former sub-stage (selection of information), the P2 components were similar under the two sleep conditions, indicating that TSD did not impair the primary attention sub-stage. In the latter sub-stage (allocation of attentional resources), the P3 components in the frontal and central regions were undermined under TSD conditions, indicating that TSD attenuated the subsequent higher-order cognitive control sub-stage. At the same time, P3 components in parietal regions were enhanced under TSD conditions, indicating the event-related compensatory neural activity triggered *via* TSD. The topographic maps (Figure 4) of P2 (100–250 ms) and P3 (250–450 ms) components also illustrate this processing. From the figure, it is clear whether the color in the frontal and central regions is lighter in TSD conditions than in the normal sleep baseline conditions and whether the color in the parietal regions is brighter in TSD conditions than in the normal sleep baseline conditions.

Kusztor et al. (2019) proposed that cognitive alterations *via* TSD include two aspects: automatic or bottom-up processing, and strategic or top-down control. The results of their study supported the notion that compared with more automated control processes, TSD was more harmful to cognitive function, which is relatively more dependent on mental effort and/or cognitive ability. The current study confirms this conclusion to some extent. Compared with the selection of information (the primary top-down processing following automatic bottom-up processing), TSD impaired the downstream cognitive control sub-stage to a higher degree. The bigger breakthrough in the current study was the finding of event-related compensatory neural activity in visual WMU processes. This may be due to the limitation of the stop-signal paradigm, which is linked to the inhibition control function that resulted in the absence of the event-related compensatory neural activity in the study by Kusztor et al. (2019). Whether TSD triggers event-related compensatory neural activity in broad cognitive functions or just in visual WMU processes is a question worth exploring in future studies.

The difference between the two tasks was helpful in understanding how event-related compensatory neural activity worked in visual WMU processes. In the current study, we employed two types of visual WMU tasks instead of only spatial or object WMU tasks. The spatial WMU task required participants to pay attention to the position where the stimulus appeared next, and the object WMU task required participants to concentrate on the specific stimulus presented. To a certain extent, the spatial WMU task was a global task and the object WMU task was a local task. Therefore, the latter task was more difficult for participants. This is supported by the significant main effect of task type, indicating that the accuracy of the spatial WMU task was higher than that of the object WMU task. The main effect of task type was significant for the P2 component, indicating that the processing of information collection in the object WMU task required more energy. In the next sub-stage, the significant main effect of task types for the P3 component was in the opposite direction, indicating that the energy required in the process of cognitive control in the object WMU task was not as high as that in the spatial WMU task. These findings are illustrated in the topographic maps (Figure 4) of the two components. In other words, event-related compensatory neural activity was more obvious in a simple task than in a more sophisticated task.

There are some limitations of the current study that deserve further consideration. First, the sample size was not very large. According to previous studies (Liu et al., 2015; Aidman et al., 2018; Honn et al., 2018; Gosselin et al., 2019; Muller-Gass and Campbell, 2019; Posada-Quintero et al., 2019; Rabat et al., 2019), the sample size of the current study was appropriate to draw a steady conclusion. Future studies can enlarge the sample size to check the generalizability of the current findings. Second, as a trade-off of the small sample size to maximize the effect of TSD, we only employed male participants to complete the experiment, which could influence the generalizability of the findings. Given that the sex difference was not related to the subject of the current study, it is necessary to explore the sex difference in another study. Third, the load of the n-back tasks was an important factor for the visual WMU function. Considering the complexity of the experiment may cause participants to be more irritable, we decided that evaluating the effects of task load in future studies was a reasonable trade-off. Fourth, the different types of stimuli—small black squares and geometric figures—raise the question of whether stimulus types or task requirements make a difference; however, this did not appear to be a significant shortcoming in the classic n-back experimental paradigm. Finally, as mentioned above, the existence of event-related compensatory neural activity in visual WMU processes after TSD is an exploratory discovery worthy of more thorough investigation and confirmation in future studies.

The current study investigated the effects of a 36-h TSD on visual WMU processes while performing spatial and object n-back tasks with simultaneous EEG recordings. The results suggest that although the indicators for selection of information (P2 components) were not affected by TSD, the indicators for allocation of attentional resources (P3 components) were significantly affected. The most significant finding was the distinct separation between the attenuation of P3 components in the frontal and central regions and the enhancement of P3 components in the parietal regions after TSD. The former indicates damaged cognitive functions in the frontal and central brain, whereas the latter is the first indication of the existence of event-related compensatory neural activity. The event-related compensatory effect provides a new perspective for understanding how sleep loss influences brain activity and the underlying mechanisms of these influences.

## Data Availability Statement

The raw data supporting the conclusions of this article will be made available by the authors, without undue reservation.

## Ethics Statement

The studies involving human participants were reviewed and approved by the Ethics Committee of the Fourth Military Medical University and the Ethics Committee of Beihang University. The patients/participants provided their written informed consent to participate in this study.

## Author Contributions

YS, JL, and XW designed the experiment. YS collected the experiment data. TS and KY analyzed the data and wrote the manuscript. LW, LX, MX, ZP, CD, HW, and TY interpreted the results of data analyses. All authors listed have read and approved the manuscript.

## Conflict of Interest

The authors declare that the research was conducted in the absence of any commercial or financial relationships that could be construed as a potential conflict of interest.

## Publisher’s Note

All claims expressed in this article are solely those of the authors and do not necessarily represent those of their affiliated organizations, or those of the publisher, the editors and the reviewers. Any product that may be evaluated in this article, or claim that may be made by its manufacturer, is not guaranteed or endorsed by the publisher.

## Footnotes

1
https://www.mathworks.com/products/matlab.html

2
https://github.com/lucklab/erplab

3
https://www.nitrc.org/projects/adjust/

4
https://letswave.cn/
